# Comparison of Estimated Excess Deaths in New York City During the COVID-19 and 1918 Influenza Pandemics

**DOI:** 10.1001/jamanetworkopen.2020.17527

**Published:** 2020-08-13

**Authors:** Jeremy Samuel Faust, Zhenqiu Lin, Carlos del Rio

**Affiliations:** 1 Brigham and Women’s Hospital, Division of Health Policy and Public Health, Department of Emergency Medicine, Harvard Medical School, Boston Massachusetts; 2 Yale New Haven Hospital, Center for Outcomes Research and Evaluation, New Haven, Connecticut; 3 Division of Infectious Diseases, Department of Medicine, Emory University School of Medicine, Atlanta, Georgia; 4 Hubert Department of Global Health, Rollins School of Public Health of Emory University, Atlanta, Georgia

## Abstract

This cohort study compares the excess deaths in New York City during the peak of the 1918 H1N1 influenza pandemic with those during the early period of the COVID-19 pandemic.

## Introduction

During the 1918 H1N1 influenza pandemic, there were approximately 50 million influenza-related deaths worldwide, including 675 000 in the US. Few persons in the US have a frame of reference for the historic levels of excess mortality currently being observed during the coronavirus disease 2019 (COVID-19) pandemic.^1^ In this study, excess deaths in New York City during the peak of the 1918 H1N1 influenza pandemic were compared with those during the initial period of the COVID-19 outbreak.

## Methods

This cohort study compared the incident rates of all-cause mortality in New York City during the peak of the 1918 H1N1 influenza pandemic and the early COVID-19 outbreak in 2020 using public data from the Centers for Disease Control and Prevention (1914-1918), The New York City Department of Health and Mental Hygiene (2020), and the US Census Bureau (2017-2020).^2,3,4,5^ This study was deemed to be exempt from institutional review approval because it used publicly availably data. This study followed the Strengthening the Reporting of Observational Studies in Epidemiology (STROBE) reporting guideline.

Analyses were performed using SAS, version 9.4 (SAS Institute). Incidence rate per person-months and corresponding 95% CIs were calculated for October and November (61 days) from 1914 through 1918, and for March 11, 2020, through May 11, 2020 (61 days), separately. Sixty-one day incident rates were divided by 2 to obtain person-month units. To compare the all-cause mortality between 2020 and 2019, an incidence rate ratio and its corresponding 95% CI was calculated.

## Results

During the peak of the 1918 H1N1 influenza outbreak in New York City, a total of 31 589 all-cause deaths occurred among 5 500 000 residents, yielding an incident rate of 287.17 deaths per 100 000 person-months (95% CI, 282.71-291.69 deaths per 100 000 person-months) (Figure, A). The incident rate ratio for all-cause mortality during the H1N1 influenza pandemic compared with corresponding periods from 1914 to 1917 was 2.80 (95% CI, 2.74-2.86). During the early period of the COVID-19 outbreak in New York City, 33 465 all-cause deaths occurred among 8 280 000 residents, yielding an incident rate of 202.08 deaths per 100 000 person-months (95% CI, 199.03-205.17 deaths per 100 000 person-months) (Figure, B). The incident rate ratio for all-cause mortality during the study period of 2020 compared with corresponding periods from 2017 through 2019 was 4.15 (95% CI, 4.05-4.24). The incident rate ratio for all-cause mortality during the peak of the 1918 H1N1 influenza pandemic and the early 2020 COVID-19 outbreak was 0.70 (95% CI, 0.69-0.72).

**Figure.**
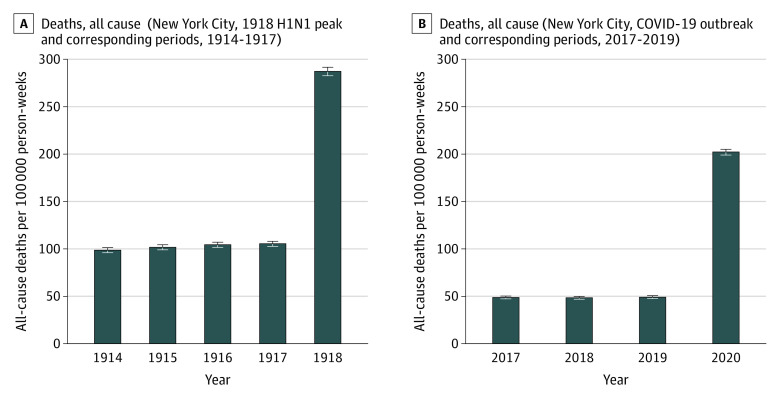
Deaths in New York City During the 1918 H1N1 Influenza Pandemic and the Coronavirus Disease 2019 (COVID-19) Pandemic and During the Preceding Years of Both Pandemics

## Discussion

This cohort study found that the absolute increase in deaths over baseline (ie, excess mortality) observed during the peak of 1918 H1N1 influenza pandemic was higher than but comparable to that observed during the first 2 months of the COVID-19 outbreak in New York City.

However, because baseline mortality rates from 2017 to 2019 were less than half that observed from 1914 to 1917 (owing to improvements in hygiene and modern achievements in medicine, public health, and safety), the relative increase during early COVID-19 period was substantially greater than during the peak of the 1918 H1N1 influenza pandemic.

One limitation of this study is that a direct comparison of the native virulence of the 1918 H1N1 influenza strain and severe acute respiratory syndrome coronavirus 2 (SARS-CoV-2) is not possible. It is unknown how many deaths due to SARS-CoV-2 infection have been prevented because of modern interventions not widely available a century ago, including standard resuscitation, supplemental oxygen, mechanical ventilation, kidney replacement therapy, and extracorporeal membrane oxygenation. If insufficiently treated, SARS-CoV-2 infection may have comparable or greater mortality than 1918 H1N1 influenza virus infection.

These findings suggest that the mortality associated with COVID-19 during the early phase of the New York City outbreak was comparable to the peak mortality observed during the 1918 H1N1 influenza pandemic. Recent polling indicates that a majority of individuals in the US believe that some states lifted COVID-19 restrictions too quickly.^6^ Specifically, shutdowns did not adequately lower caseloads in many areas, meaning that subsequent spikes in new cases during the summer stretched US hospital resources in many areas. We believe that our findings may help officials and the public contextualize the unusual magnitude of the COVID-19 pandemic, leading to more prudent policies that may help to decrease transmission by decreasing the effective reproduction number of SARS-CoV-2 and prevent the exhaustion of essential supplies of life-saving resources in the coming weeks and beyond.
