# Error in the Figure

**DOI:** 10.1001/jamanetworkopen.2020.21496

**Published:** 2020-09-09

In the Research Letter, “Comparison of Estimated Excess Deaths in New York City During the COVID-19 and 1918 Influenza Pandemics,”^1^ published on August 13, 2020, in the Figure, A and B, the y-axis label should have been all-cause deaths per 100 000 person-months, not person-weeks. This article has been corrected.
